# Characterization of a Novel Hepatitis C Subtype, 6xj, and Its Consequences for Direct-Acting Antiviral Treatment in Yunnan, China

**DOI:** 10.1128/spectrum.00297-21

**Published:** 2021-08-25

**Authors:** Yuanyuan Jia, Wei Yue, Qinghua Gao, Rui Tao, Yaxiang Zhang, Xiaoyang Fu, Yang Liu, Li Liu, Yue Feng, Xueshan Xia

**Affiliations:** a Faculty of Life Science and Technology, Kunming University of Science and Technology, Kunming, China; b Department of Infectious Diseases and Liver Diseases, The First People’s Hospital of Yunnan Province, Kunming, China; Johns Hopkins Hospital

**Keywords:** hepatitis C virus, DAA, follow-up, RAS, novel subtype, resistance-associated substitutions

## Abstract

Hepatitis C virus (HCV) has a high rate of genetic variability, with eight genotypes and 91 subtypes. The genetic diversity of HCV genotype 6 (HCV-6) is the highest with 31 subtypes, and this genotype is prevalent in Southeast Asia. In this study, we investigated 160 individuals with chronic hepatitis C in Yunnan Province, China. Using reverse transcription (RT)-PCR and Sanger sequencing, 147 cases were successfully amplified and genotyped as 3b (4.9%), 3a (19.73%), 6n (12.24%), 1b (7.48%), 2a (6.12%), 6a (2.04%), 1a (0.68%), 6v (0.68%), and 6xa (0.68%), with eight sequences remaining unclassified. Subsequently, the eight nearly full-length genomes were successfully amplified and analyzed. The eight complete coding sequences formed a phylogenetic group that was distinct from the previously assigned HCV-6 subtypes and clustered with two previously unnamed HCV-6 sequences. Furthermore, Simplot analysis showed no recombination and the *p*-distance was more than 15% in comparison to the 6a to 6xi subtypes. Taken together, we identified a new HCV-6 subtype, 6xj, which originated approximately in 1775 according to Bayesian analyses. Moreover, all eight individuals received follow-up assessments at 44 weeks from the beginning of their 12-week treatments of sofosbuvir/velpatasvir (after-treatment week 32). One case relapsed at after-treatment week 32. Next-generation sequencing (NGS) was conducted and showed that the treatment failure case had two suspected antiviral resistance mutations, NS5A V28M (a change of V to M at position 28) and NS5B A442V, compared with the baseline. Overall, this newly identified 6xj subtype further confirmed the high diversity of the HCV-6 genotype. The newly identified resistance-associated amino acid substitutions may help inform future clinical treatments.

**IMPORTANCE** This study investigated the genetic diversity of hepatitis C virus (HCV), particularly in relation to genotype 6, which is prevalent in Yunnan, China, and is often difficult to treat successfully. We identified a new HCV-6 subtype, 6xj, which is an ancient strain. Moreover, all eight individuals with the novel subtype received follow-up assessments at 44 weeks from the beginning of their treatments. One case relapsed after 8 months of withdrawal. NGS was conducted and showed that the isolate from the treatment failure case had two suspected antiviral resistance mutations, NS5A V28M and NS5B A442V, compared with the baseline. Overall, this newly identified 6xj subtype further confirmed the high diversity of the HCV-6 genotype. The newly identified resistance-associated amino acid substitutions may help inform future clinical treatments. We believe that our study makes a significant contribution to the literature based on the results described above.

## INTRODUCTION

Hepatitis C virus (HCV), from the genus *Hepacivirus* in the family *Flaviviridae*, is a positive-sense single-stranded RNA (ssRNA) virus. Its genome encodes a polyprotein in a single open reading frame (ORF) that is approximately 9,600 nucleotides (nt) in length. Persistent HCV infection can lead to chronic hepatitis C, cirrhosis, hepatocellular carcinoma, and even death (1). According to the Global Hepatitis Report by the World Health Organization (WHO), HCV infected approximately 71 million people (1%) worldwide as of 2015, and an estimated 1.75 million new infections occur every year (2), leading to a severe global burden of liver disease.

HCV displays high genetic diversity as a result of defects in the repair activity of RNA-dependent RNA polymerase and the absence of 5′-to-3′ exonuclease activity (3). Based on phylogenetic analysis of the genome sequence, HCV is now classified into eight genotypes (GTs) and 91 subtypes (4, 5). These genotypes are characterized as having greater than 30% divergence in the complete genome sequence, and the subtypes diverge by more than 15% (6). The geographical distributions of these genotypes also differ. HCV genotype 1 (HCV-1 or HCV GT1), HCV-2, and HCV-3 are associated with worldwide epidemics, whereas genotypes 4 to 8 are endemic. For instance, HCV-4 is predominant in the Middle East and Africa, genotype 5 in Southern Africa (7), genotype 6 in southern China and Southeast Asia, and genotype 7 in central Africa (8). More recently, genotype 8 has been identified in India (9). Among the eight HCV genotypes, genotype 6 exhibits a high degree of genetic complexity and diversity, with 31 subtypes confirmed by the International Committee on Taxonomy of Viruses (ICTV) (4, 5). In China, HCV genotype 6 is common; subtypes 6a, 6e, 6n, 6v, 6w, 6xa, 6xe, 6xh, and 6xi were first identified and named in China over the past 20 years (4, 5). Yunnan is the main hot spot for identifying new HCV-6 subtypes, with 6n, 6v, 6xa, 6xe, and 6xi reported so far (4, 5).

To date, accumulating evidence has shown that direct-acting antiviral (DAA) treatment regimens used for patients with chronic HCV infections are very safe and ensure high rates of sustained virologic responses (SVR) (>90%) (10). A major breakthrough in the DAA therapy for chronic HCV infection has been the development of pangenotype drug combination regimens approved for the treatment of adults infected with HCV genotypes 1 to 6, including ledipasvir plus sofosbuvir, sofosbuvir plus daclatasvir, sofosbuvir plus velpatasvir, and glecaprevir plus pibrentasvir (11). However, virologic failure following DAA therapy is an unfortunate event that can occur for all HCV genotypes and in various clinical situations. An important reason for this relates to resistance-associated amino acid substitutions (RASs), which lead to resistance-associated viral variants (RAVs) (12). Previous studies have reported the corresponding RASs of HCV GT1a, GT1b, GT2, GT3, and GT4 in the NS3, NS5A, and NS5B regions (13). To date, only limited data on the RASs of HCV genotypes 5 and 6 are available. However, some studies have reported the occurrence of resistance in HCV-6 and differences between this and other genotypes, with HCV-6 RASs mainly associated with the following positions: NS3 41, 43, 56, 156, and 168; NS5A 24, 28, 31, 32, 58, 92, and 93; and NS5B 282 (14–16).

Yunnan is located in the southwestern border of China and has diverse HCV-6 subtypes. In the current study, we explored the epidemic and genotypic distribution of treatment-naive chronic hepatitis C patients in Yunnan Province and characterized a new HCV subtype designated 6xj. Moreover, therapeutic efficacy and the resistance variants of the 6xj subtype were also investigated in follow-up assessments of individuals.

## RESULTS

### Demographic characteristics.

In the present study, a total of 160 chronic hepatitis C patients were recruited from Yunnan, China. The demographic characteristics of the 160 subjects are summarized as follows. The mean age ± standard deviation of the participants was 42.5 ± 13.1 years, and the ratio of males to females was 100:60. The following clinical characteristics were identified: mean alanine transaminase (ALT), 59.1 ± 85.0 IU/liter; mean aspartate transaminase (AST) 57.6 ± 83.2 IU/liter; and mean HCV RNA, 5.3 ± 1.1 log_10_ IU/ml.

### Distribution of HCV genotypes and subtypes.

**FIG 1:**
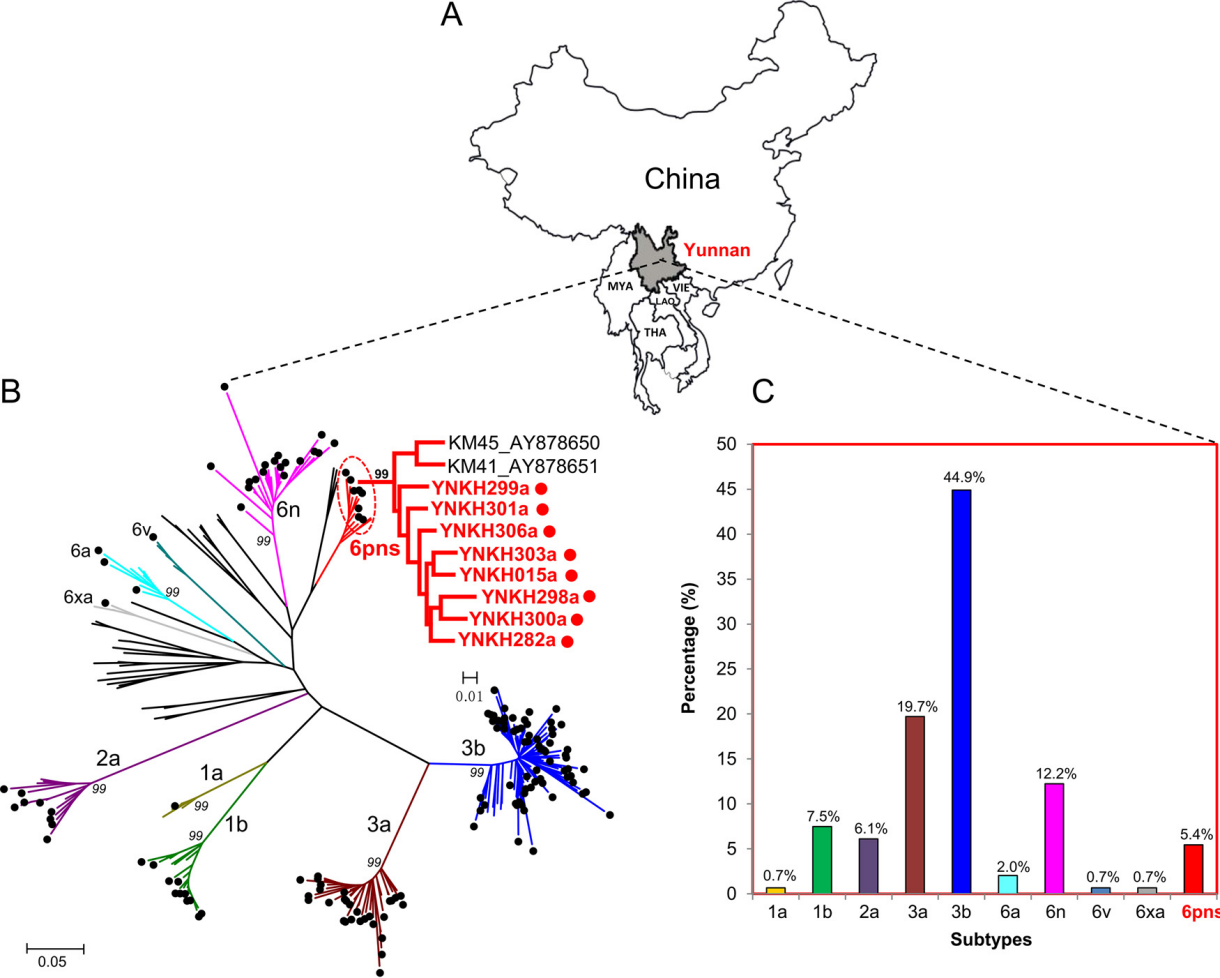
Map of the study region and phylogenetic tree analysis and genotypic distribution of HCV isolates among individuals with chronic hepatitis C in Yunnan, China. (A) Map of the study region. The Yunnan province of southwestern China is marked by shading. MYA, Myanmar; THA, Thailand; LAO, Laos; VIE, Vietnam. (B) Phylogenetic tree based on the partial NS5B sequences amplified from 147 HCV-infected individuals. The different subtypes are shown in different colors. (C) Comparison of HCV subtype distribution rates among individuals with chronic hepatitis C in Yunnan, China.

Among 160 patients with chronic hepatitis C, 147 partial NS5B gene fragments were successfully amplified and sequenced, with a success rate of 91.9% (147/160). The failure of amplification in 13 cases was possibly due to low viral loads (2.9 ± 0.5 log_10_ IU/ml) and weak primer specificity. Phylogenetic analyses were conducted based on the NS5B fragments of HCV (Fig. 1B). Our results indicated that 3b was the most common subtype (44.9%, 66/147), followed by 3a (19.7%, 29/147), 6n (12.2%, 18/147), 1b (7.5%, 11/147), 2a (6.1%, 9/147), 6a (2.0%, 3/147), and 1a, 6v, and 6xa (each of the last three subtypes occurring at 0.7% or 1/147) (Fig. 1C). The remaining eight strains were classified as genotype 6. These remaining eight isolates, in combination with the previously identified strains KM41 and KM45 (6), clustered separately from known HCV-6 subtypes and formed a distinct, novel cluster with a 99% bootstrap value, suggesting the presence of a new HCV-6 subtype (Fig. 1B).

### Identification and characterization of the novel HCV subtype.

**FIG 2:**
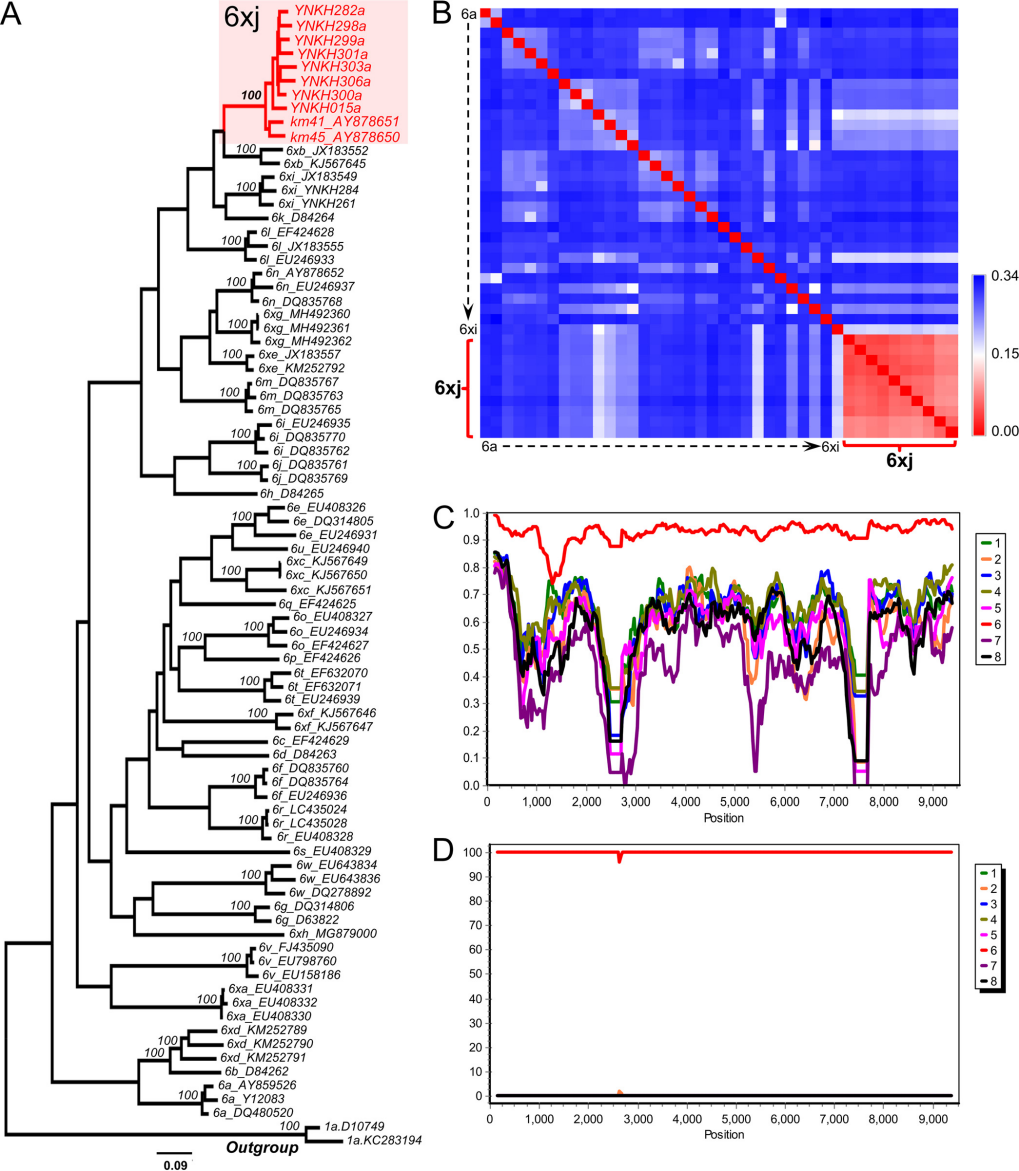
Phylogenetic tree and analyses of the nucleotide divergence between 6xj and the other known genotype 6 subtypes (6a to 6xi) and recombinant based on full-length HCV genome sequences. (A) Previously reported HCV subtype 6 reference sequences (6a to 6xi) were used. Phylogenetic analysis was performed by the maximum-likelihood method, based on the GTR+G+I substitution model, with 1,000 bootstrap replicates, using MEGA version 6. The HCV 6xj sequences are marked in red. (B) Pairwise comparisons of nucleotide similarities between 3 HCV 6xi strains and 30 reference genotype 6 sequences. (C) Analysis using the SimPlot program demonstrates the genetic distance to the reference strain in different parts of the genome. (D) Bootscan plot using Simplot 3.5.1 software based on 100 replicates with a 300-bp sliding window moving in steps of 50 bases demonstrates the phylogenetic relationship to the reference strain.

We successfully characterized the nearly full-length genome sequences of unclassified HCV isolates from eight patients, each with 12 overlapping fragments. Their genomes were 9,431 nucleotides (nt) long, starting from the 5′ untranslated regions (UTRs). Eight genomes shared common sizes in terms of their complete ORFs (9,045 nt or 3,015 amino acids [aa]) and in the 10 protein-coding regions. These contain the core (573 nt or 191 aa), E1 (576 nt or 192 aa), E2 (1,092 nt or 364 aa), P7 (189 nt or 63 aa), NS2 (651 nt or 217 aa), NS3 (1,893 nt or 631 aa), NS4A (162 nt or 54 aa), NS4B (783 nt or 261 aa), NS5A (1,353 nt or 451 aa), and NS5B (1,776 nt or 591 aa) regions. Furthermore, based on the eight complete coding sequences obtained and the two previously reported sequences (KM41 and KM45) (6), a maximum-likelihood (ML) tree was reconstructed with the inclusion of subtypes 6a to 6xi. The 10 strains grouped, with a strong bootstrap support of 100%, into a distinct monophyletic clade that was closest to subtype 6xb (Fig. 2A).

Pairwise distances were calculated between the nucleotide sequences of the 10 full-length genomes, as well as between the potential new HCV-6 subtype and subtypes 6a to 6xi. The results showed that the nucleotide distances were less than 15% among the 10 sequences, while there was a greater than 15% distance between the sequence of the potential new HCV-6 subtype and those of the other subtypes, 6a to 6xi (Fig. 2B). The strains were isolated from 10 HCV-infected patients and included eight strains from this study and two from a previous study without obvious epidemiological linkage to Yunnan. Moreover, no recombination breakpoints were detected within the 10 sequences (Fig. 2C and D). Taken together, our findings met the criteria for a new HCV subtype, and we tentatively designated these strains as constituting the novel subtype 6xj (KM41, KM45, YNKH015a, YNKH282a, YNKH298a, YNKH299a, YNKH300a, YNKH301a, YNKH303a, and YNKH306a, in alphabetical order).

### Evolutionary history of HCV subtype 6xj.

**FIG 3:**
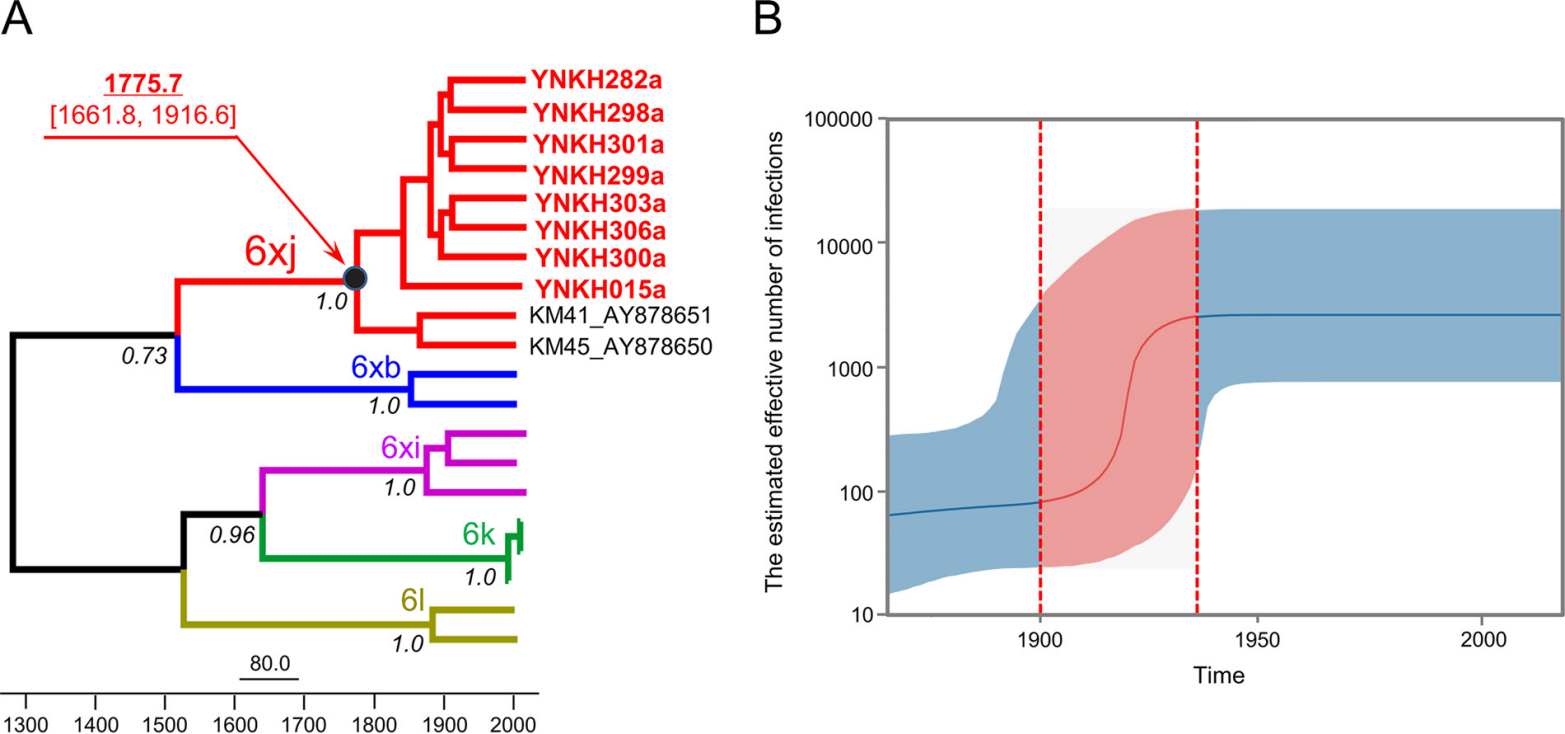
Maximum clade credibility (MCC) tree of HCV full-length coding region sequences and Bayesian skyline plot (BSP) estimating the past population dynamics of HCV 6xj. (A) The MCC tree was constructed by Bayesian Markov chain Monte Carlo (MCMC) analysis based on HCV complete coding region sequences. HCV 6xj strains from Yunnan are highlighted in red. The node of the HCV subtype 6xj clade is shown by the black dot. The node age with 95% confidence interval is shown at top left. (B) The BSP was reconstructed using Tracer version 1.7.1. The *y* axis represents the estimates of the effective numbers of HCV 6xj strains, and the *x* axis represents time. The solid line represents the median estimates, and the shaded area represents the 95% confidence intervals. The pink area represents a period of exponential growth.

Based on the full-length genome, including the 10 sequences involved in this study, Bayesian molecular clock analyses were performed to estimate the time to the most recent common ancestor (tMRCA). Taking all of the 6xj sequences as a single lineage, the estimated tMRCA was dated to 1775.7 (95% highest probability density [HPD], 1661.8, 1916.6) (Fig. 3A). Simultaneously, the 10 genomes clustered with the two 6xb sequences, and they collectively formed a group that included the subtypes 6xb and 6xj and shared a common ancestor (Fig. 3A). In addition, the Bayesian skyline plot (BSP) illustrates the demographic history of 6xj: the effective population size increased slowly from 1865 to 1900. However, this changed to fast exponential growth until the year 1930, followed by stabilization in population size that continues to this day (Fig. 3B).

### RASs of the HCV subtype 6xj.

**FIG 4:**
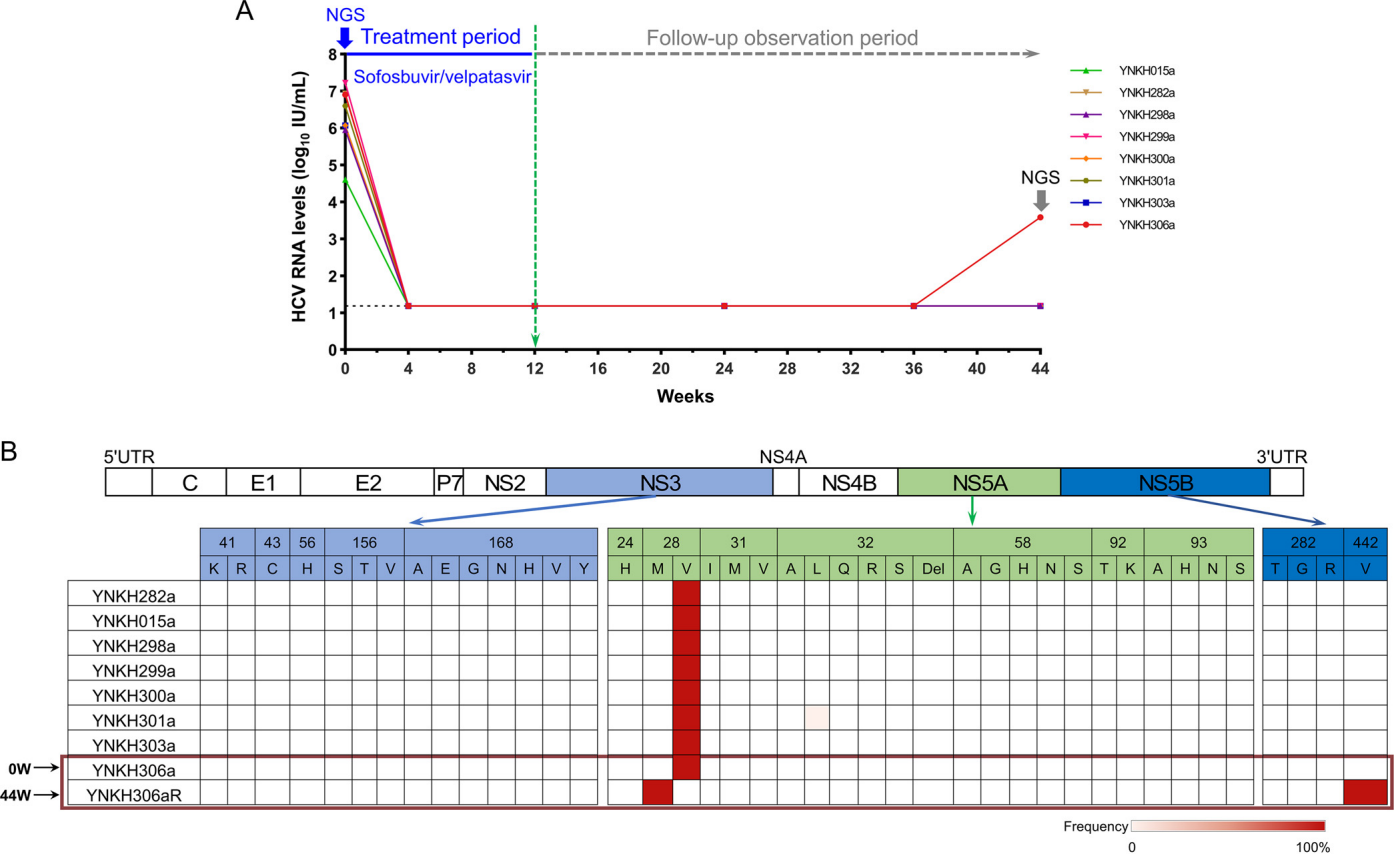
Changes of HCV RNA levels and resistance-associated substitutions (RASs) among the eight chronic hepatitis C patients infected by the new HCV 6xj subtype during sofosbuvir/velpatasvir (SOF/VEL) treatment. (A) Patients infected with HCV subtype 6xj received SOF/VEL (Epclusa) (FDC, 400/100 mg per tablet; Gilead Sciences) at 1 tablet once daily for 12 weeks. Serum HCV RNA levels were assessed at on-treatment weeks 0, 4, and 12 and at after-treatment weeks 12, 24, and 32 (follow-up weeks 24, 36, and 44). The lowest detection limit for HCV RNA was 1.2 log_10_ IU/ml (15 IU/ml). (B) RASs of the eight baseline sequences (week 0 [0W]) and one treatment failure sequence at after-treatment week 32 (follow-up week 44 [44W]) analyzed in the NS3, NS5A, and NS5B regions by next-generation sequencing (NGS).

The demographic characteristics and clinical characteristics of the eight new HCV-6 subtype isolates are shown in Fig. 4A and in Table S1 in the supplemental material. To further characterize the new HCV subtype genomes, the RASs of NS3, NS5A, and NS5B proteins were analyzed using next-generation sequencing (NGS). By consulting and summarizing the research on HCV genotype 6 RASs, we analyzed the eight 6xj sequences. Strikingly, all eight patients with HCV 6xj infection having the 28V substitution in NS5A was present in 100% of the viral population at baseline, and only the YNKH301a containing the 32L mutation in NS5A was present in 2% (Fig. 4B). In addition, all eight patients adopted the treatment regimen of sofosbuvir/velpatasvir (Epclusa) (fixed-dose combination [FDC], 400/100 mg per tablet; Gilead Sciences) for 12 weeks. After follow-ups at 44 weeks after the beginning of treatment with sofosbuvir/velpatasvir (after-treatment week 32), the patient infected with HCV strain YNKH306a, a 42-year-old man, had a baseline HCV RNA measurement of 6.9 log_10_ IU/ml. This patient was administered the sofosbuvir/velpatasvir regimen for 12 weeks, during which he had an HCV RNA measurement of <1.2 log_10_ IU/ml (15 IU/ml). Unfortunately, at after-treatment week 32, his ALT had increased to 189 IU/liter, his AST was abnormal at 92 IU/liter, and his HCV RNA measurement was 3.58 log_10_ IU/ml, indicating that he had relapsed (Fig. 4A). To further explore the cause of the relapse associated with strain YNKH306a, both treatment-naive and treatment failure sequences involving HCV NS3, NS5A, and NS5B regions were successfully amplified by an NGS method. We compared the two sequences for all amino acid substitutions within the regions that were targets of the DAAs employed and found two divergent amino acid substitutions, V28M in the NS5A protein and A442V in the NS5B protein (Fig. 4B).

## DISCUSSION

In the present study, we evaluated the HCV genotype and subtype distributions among 160 patients with chronic hepatitis C in Yunnan, China. Our results demonstrated that the predominant subtypes in Yunnan Province in southwest China were 3a and 3b (64.6%), followed by genotype 6n (12.2%), which is consistent with previous findings (17). Most notably, many studies have documented that patients with chronic HCV genotype 3 infections exhibit more rapid progression of hepatic fibrosis, cirrhosis, and hepatocellular carcinoma (18) and are less responsive to current direct-acting antiviral regimens than patients infected with other genotypes (19). This undoubtedly poses a great challenge to the prevention and treatment of HCV genotype 3 in Yunnan, China.

The most important finding of this study was the identification of a novel HCV subtype, tentatively designated 6xj. This new subtype met the following principal criteria proposed by the ICTV: (i) a highly supported monophyletic cluster based on the analyses of complete genome sequences, (ii) a mean intersubtype nucleotide divergence of between 15% and 30% compared with representative full-length genome sequences of all known HCV-6 subtypes, (iii) strains obtained from individuals with no epidemiologic link, and (iv) no significant evidence of recombination (6).

Research has shown that most HCV-6 subtypes have first been identified in Southeast Asia, including Vietnam (6d, 6h, 6k, 6t, 6u, 6xb, 6xc, and 6xf), Thailand (6b, 6c, 6f, 6i, 6j, and 6m), Laos (6xd), Myanmar (6xg), and Indonesia (6g) (4, 5), suggesting that the Southeast Asian region is an epicenter of HCV-6 subtype epidemics. To date, nine HCV-6 subtypes have first been identified in China, including 6a, 6e, 6n, 6v, 6w, 6xa, 6xe, 6xh, and 6xi. Of these, 6n, 6v, 6xa, 6xe, and our recently reported 6xi were first detected in Yunnan, which is located in southwestern China and borders the opium-producing “Golden Triangle” region composed of Myanmar, Laos, Thailand, and Vietnam (Fig. 1a). The transmission of several human viruses is closely associated with illegal drug trafficking (20, 21). The prevalence of illegal drugs may result in an increase in intravenous drug users (IDUs) due to high drug trafficking rates (22). Among all the HCV-6 subtypes, the 6e, 6n, 6w, 6xa, 6xe, and 6xg subtypes were first reported in IDUs. HCV 6xj, named in the present study, is also characterized among IDUs in Yunnan, China. Presumably, the geographical location of Yunnan has also increased the prevalence of the extremely complex and diverse HCV-6 subtypes.

HCV subtypes have special epidemiological significances, which are used to trace transmission and evolution. In contrast with studies based on partial genome regions (which showed that some of the HCV-6 subtypes may have originated in the 20th century) (6, 23), we conducted Bayesian phylogenetic analyses based on the complete coding region. As shown in the maximum clade credibility (MCC) tree constructed with the complete coding sequences, the common ancestor of the 6xj subtype cluster dates to approximately 1775. This is in accordance with previous studies which found that the tMRCA of the complete coding region from the HCV-6 genotype dates back 300 to 400 years (1, 24). Previous studies have also indicated that the HCV-6 genotype is the oldest lineage (1, 24). In addition, according to the Bayesian phylogenetic tree, the 10 new 6xj genomes clustered with two previously reported 6xb sequences isolated from individuals in Vietnam. In terms of nucleotide sequence similarity and phylogeny, the 6xj nucleotides were 85.4 to 85.5% similar to those of subtype 6xb. Both results show that 6xj and 6xb originate from the same HCV lineage and have a common ancestor.

To date, DAAs used for HCV-6 treatment mainly include pangenotype regimens (11), as well as a special regimen of sofosbuvir and ledipasvir for a specific HCV-6 subtype (25). A number of studies have shown that DAAs have higher sustained virologic response (SVR) rates for genotype 6, reaching 83.3% to 100% (25). However, other studies have also shown that genotype 6 still induces a high treatment failure rate. One study showed that SVR12 rate (sustained virological response at 12 weeks, recognized as the measure of treatment success, defined as undetectable HCV RNA in the blood at the end of treatment), following treatment with sofosbuvir and ledipasvir, was only 64.1% (26). Combined with liver cirrhosis, it was 41%, and ultimately, 19.3% of patients relapsed following the cessation of treatment (26). Antiviral resistance has been a critical issue in the treatment of HCV, and retreatment options after initial treatment failure are important considerations. Antiviral resistance and treatment failure are usually related to the presence of RAVs. The drug resistance amino acid substitutions most commonly observed in patients with HCV genotype 6 infections are 41K/R (a change to K or R at position 41), 43C, 56H, 156S/T/V, and 168A/E/G/N/H/V/Y in NS3, 24H, 28M/V, 31I/M/V, 32A/L/Q/R/S/Del, 58A/G/H/N/S, 92T/K, and 93A/H/N/S in NS5A, and 282T/G/R in NS5B (14–16). Compared with these known RAVs, all 10 HCV 6xj strains in this study naturally possessed the substitution of 28V in NS5A, and one isolate (YNKH301a) had a 32L variant in the NS5A protein, whereas SVR44 rate was achieved by 87.5% of patients (7/8), indicating that HCV 6xj strains naturally contain the two substitutions 28V and 32L in NS5A, which may be not the main RAVs. Notably, our results indicate that strain YNKH306aR, isolated from a relapsed case at after-treatment week 32, had two divergent amino acid substitutions, V28M in the NS5A protein and A442V in the NS5B protein, compared with the sequences from baseline sample. These two amino acid sites are located in the key regions of the NS5A phosphoprotein and NS5B RNA-dependent RNA polymerase, respectively, which are key viral enzymes that direct-acting antivirals specifically block. Thus, we speculated that the two mutations V28M in NS5A and 442V in NS5B may be the key drug resistance mutations related to virus rebound in this patient. In addition, our results highlight that investigating the presence of NS5A and NS5B RASs before initiation of DAAs for hepatitis C virus is of great significance for tailoring treatment regimens. Meanwhile, during DAA therapy, the evolution of RAVs is now one of the most critical challenges in the clinical treatment of HCV infection.

There were some limitations to this study. There are only 8 HCV 6xj subjects with follow-up, a very small study number. The small amount of treatment data is interesting but too limited to guide decision-making on its own. It is necessary to strengthen continuous molecular screening of HCV, as well as long-term follow-up epidemiological surveys and direct-acting antiviral treatment aimed at HCV 6xj subtypes in Yunnan, China. Another limitation of the study is that the pathogenicity of the two suspected drug resistance mutations NS5A V28M and NS5B A442V is not very clear, and further functional verification at the cellular level is needed.

In conclusion, we characterized a new HCV subtype, 6xj, in Yunnan, China. Bayesian analysis showed that 6xj was an ancient strain. By tracking treatment failure cases, two suspected RAVs, NS5A V28M and NS5B A442V, were identified. These findings again emphasize that Yunnan Province is a hot spot for HCV-6 infection, and our research provides essential reference data for the clinical treatment of 6xj. All the factors we examined are of great importance in epidemiological investigations and the monitoring of drug resistance.

## MATERIALS AND METHODS

### Ethical statement.

The study was approved by the First People’s Hospital of Yunnan Province Ethics Committee. Written informed consent was obtained from all participants prior to the study.

### Study population.

In this study, serum samples were collected between January 2018 and October 2018 from 160 chronic hepatitis C individuals who lived in Kunming city in Yunnan, China (Fig. 1A). All participants were diagnosed with hepatitis C for the first time and had not undergone any treatment. Demographic information relating to age and gender were obtained via self-report questionnaires. Clinical parameters of disease progression, including alanine aminotransferase (ALT) and aspartate aminotransferase (AST) levels and HCV RNA viral loads, were determined at the time of sampling. Plasma was separated from whole blood samples using EDTA tripotassium salt and stored at −80°C for HCV RNA extraction.

### RNA extraction, HCV gene amplification, and sequencing.

HCV RNA was isolated from 200-μl plasma samples using the MiniBest viral RNA/DNA extraction kit according to the procedure described in the manual. The NS5B region was used to determine the HCV genotype and subtype, based on NS5B of strain H77 (nucleotides 8266 to 9303). Sequences were amplified by nested PCR using the PCR primers and conditions reported in a previous study (5). To confirm the novel subtype, complete HCV sequences from 12 overlapping HCV genomic fragments were amplified using reverse transcription (RT)-nested PCR as reported previously (5). Amplified PCR products were detected by electrophoresis on a 1.0% agarose gel under UV illumination and purified using a DNA purification kit. The products were sequenced by Tsingke Biological Technology Co. on an ABI 3730XL automated DNA sequencer.

### Sequence analyses.

Sequencing data were initially verified using the National Center for Biotechnology Information (NCBI) Basic Local Alignment Search Tool (BLAST). Reference sequences in GenBank were used for a comparative analysis of all HCV genomic sequences. Multiple alignments of the selected sequences were performed using Clustal version 1.8.1 software. The data generated were processed using BioEdit version 7.1.5 software. Phylogenetic trees based on the data sets obtained were constructed with the maximum-likelihood method using MEGA version 6.0.6 and the general time reversible plus gamma distribution plus invariant sites (GTR+G+I) model. Bootstrap values were calculated based on 1,000 replications of the alignment. Genetic pairwise distance was calculated using MEGA version 6.0.6. The SimPlot and Bootscan analyses were performed by using the SimPlot program, with a sliding window of 300 bp and 50-base steps.

To explore the evolutionary history of the new HCV subtype in China, the nearly full-length genome sequences with known sampling years and sampling locations were subjected to Bayesian analysis. Bayesian coalescent analysis using the Markov chain Monte Carlo (MCMC) sampling method was performed through BEAST version 1.7.5 under the uncorrelated log-normal relaxed clock model with the GTR+G+I nucleotide substitution model, a coalescent Bayesian skyline plot tree prior, and a relaxed uncorrelated lognormal molecular clock model. Each MCMC analysis was executed for 30 million states, with sampling at every 30,000 states. The model with an effective sampling size (ESS) of >200 was selected. Posterior probability densities were determined in Tracer version 1.7.1, and 10% of each chain was discarded as burn-in. The maximum clade credibility (MCC) tree was summarized with Tree Annotator version 1.7 and scanned using FigTree version 1.4.0. In addition, population dynamics were constructed under a coalescent Bayesian skyline plot tree prior and a piecewise linear skyline model with 10 groups using BEAST version 1.7.5. The Bayesian skyline plot was reconstructed using Tracer version 1.7.1.

RASs of the NS3, NS5A, and NS5B regions were analyzed using Sanger sequencing data. To further investigate the baseline sequence and the relapse-associated RASs of the potential new HCV-6 subtype, next-generation sequencing (NGS) was performed. The resulting binary sequence alignment/Map data were visualized by using the Integrative Genomics Viewer software and subsequently converted into variant call format files to view the RASs of the NS3, NS5A, and NS5B regions.

### Data availability.

The nucleotide sequences reported in this study have been submitted to GenBank with accession numbers MZ171127 to MZ171134 for this complete HCV genome.
