# Supplementary material for: Characterization of a Novel Hepatitis C Subtype, 6xj, and Its Consequences for Direct-Acting Antiviral Treatment in Yunnan, China
Source: Microbiol Spectr. 2021 Aug 25;9(1):10.1128/spectrum.00297-21. doi: 10.1128/spectrum.00297-21 (PMC8552672; doi:10.1128/spectrum.00297-21)
Supplement: SUPPLEMENTAL FILE 1 — Supplemental material. Download SPECTRUM00297-21_Supp_1_seq8.pdf, PDF file, 0.02 MB [file spectrum00297-21_supp_1_seq8.pdf]

Table S1. Baseline demographic and clinical characteristics of eight new HCV-6xj subtype infected patients

Isolate	Age	Gender	ALT(IU/L)	AST(IU/L)	HCV-RNA(log_{10} IU/mL)
YNKH015a	NA	F	NA	NA	4.6
YNKH282a	36	F	NA	NA	6.9
YNKH298a	50	M	759	808	6.0
YNKH299a	55	F	18	29	7.2
YNKH300a	53	F	92	87	6.1
YNKH301a	32	M	59	40	6.6
YNKH303a	44	M	39	41	6.1
YNKH306a	42	M	263	223	6.9

ALT: Alanine aminotransferase; AST: Aspartate aminotransferase; F: Female; M: Male;
NA: Not appropriate
